# Characteristics and Safety of Consumer Chatbots for Emergent Adolescent Health Concerns

**DOI:** 10.1001/jamanetworkopen.2025.39022

**Published:** 2025-10-23

**Authors:** Ryan C. L. Brewster, Aydin Zahedivash, Gabriel Tse, Florence Bourgeois, Scott E. Hadland

**Affiliations:** 1 Department of Neonatology, Beth Israel Deaconess Medical Center, Boston, Massachusetts; 2 Department of Pediatrics, Boston Children’s Hospital, Boston, Massachusetts; 3 Department of Pediatrics, Stanford University School of Medicine, Stanford, California; 4 Harvard-MIT Center for Regulatory Science, Harvard Medical School, Boston, Massachusetts; 5 Pediatric Therapeutics and Regulatory Science Initiative, Computational Health Informatics Program, Boston Children’s Hospital, Boston, Massachusetts; 6 Division of Adolescent and Young Adult Medicine, Mass General for Children, Boston, Massachusetts; 7 Department of Pediatrics, Harvard Medical School, Boston, Massachusetts

## Abstract

This cross-sectional study examines content policies and assesses behaviors of consumer chatbots in response to adolescent health crises.

## Introduction

Generative artificial intelligence (AI) chatbots supported by large language models (LLMs) are capable of sophisticated, human-like conversations across a range of applications. Adolescents are increasingly engaging with chatbots for social connection and may disclose emergent health issues, such as suicidal ideation.^1,2^ However, few regulations govern the appropriate use of these technologies, resulting in safety risks and lawsuits against chatbot companies.^3^ To further elucidate potential harms, we aimed to examine the content policies of consumer chatbots and assess behaviors of consumer chatbots in response to adolescent health crises.

## Methods

The cross-sectional study was exempted from the Boston Children’s Hospital institutional review board, and informed consent was not required because it did not involve human participants. We followed the STROBE reporting guideline. We identified the most visited consumer chatbots, classified as companion and general-assistant platforms (eTable 1 in Supplement 1). We excluded those with pornographic content or developed explicitly for mental health. Additional details on cohort selection appear in eMethods in Supplement 1. We analyzed relevant policies and terms of service (eg, minimum age of use) from publicly available documentation, including explicit provisions addressing hate speech, sexual content, violence, and self-harm. Categories were derived inductively based on documentation review.

We conducted a red teaming exercise, in which chatbots are systematically probed to expose vulnerabilities or inappropriate behaviors, to assess chatbot performance across emergent adolescent concerns.^4^ We used patient vignettes for suicidal ideation, sexual assault, and substance use adapted from validated medical education case studies (eTable 2 in Supplement 1). One investigator (R.B.) interacted with the study chatbots using the persona and standardized prompts derived from each vignette. Transcripts of the chatbot conversations were evaluated to measure empathy, understandability, and clinical appropriateness using 3-point Likert scales (eTable 3 in Supplement 1). Recognition of the need for care escalation and provision of resource referral was assessed as a binary variable. Two board-certified pediatricians (G.T. and A.Z.), masked to the chatbot source, independently applied the rubric and discussed discrepancies to reach consensus. We compared the proportion of responses rated 3 (highest) or yes for each domain between companion and general-assistant chatbots with the Fisher exact test, considered significant at *P* < .05. Data were analyzed from March 1 to July 1, 2025 using R version 4.3.1 (R Project for Statistical Computing).

## Results

This study examined 25 chatbots, and the transcripts of 75 conversations. Most chatbots were companion platforms (15 [60%]) with a median of 9 600 000 (IQR, 1 700 000-201 800 000) monthly visits (Table). Nine chatbots (36.0%) had age verification procedures, and 14 (56.0%) required user sign-in. Compared with companion chatbots, general-assistant chatbots more commonly included content policies for self-harm (7 [46.7%] vs 10 [100%]; *P* = .008).

**Table.** Chatbot Characteristics and Policies, by Chatbot Type

Characteristic	Chatbot, No. (%)		
	Companion chatbots (n = 15)	General-assistant chatbots (n = 10)	Overall (N = 25)
Monthly visits, median (IQR), millions	1.8 (1.2-19.8)	152.35 (13.0-277.1)	9.6 (1.7-201.8)
Response format types			
Text	14 (93.3)	10 (100.0)	24 (96.0)
Voice	5 (33.3)	1 (10.0)	6 (24.0)
Images	10 (66.7)	9 (90.0)	19 (76.0)
Sign-in required	8 (53.3)	6 (60.0)	14 (56.0)
Minimum age, y^a^			
13	4 (26.7)	6 (60.0)	10 (40.0)
14	1 (6.7)	0	1 (4.0)
17	1 (6.7)	0	1 (4.0)
18	9 (60.0)	4 (40.0)	13 (52.0)
Parental consent policy^b^	4 (26.7)	6 (60.0)	10 (40.0)
Age verification^c^	5 (33.3)	4 (40.0)	9 (36.0)
Harmful content policies^d^			
Hate speech	12 (80.0)	9 (90.0)	21 (84.0)
Sexual	8 (53.3)	9 (90.0)	17 (68.0)
Violence	11 (73.3)	9 (90.0)	20 (80.0)
Self-harm	7 (46.7)	10 (100.0)	17 (68.0)

^a^ Specified minimum age for use of service.

^b^ Stated policy requiring consent of parents for users below minimum age.

^c^ Standard process for verifying a user’s age to ensure they meet minimum age requirements; for example, entering their date of birth.

^d^ Hate speech refers to language that attacks or uses discriminatory language with reference to a person or identity group. Sexual content refers to language related to anatomical organs and genitals, romantic relationships and sexual acts, including vulgar content, prostitution, pornography, and abuse. Violence refers to language related to physical actions intended to hurt, injure, damage, or kill someone or something. Self-harm refers to language related to physical actions intended to purposely hurt, injure, damage one’s body, or kill oneself.

On average, chatbot responses received the highest rating for understandability (61 [81.3%]) and empathy (47 [62.7%]), while 35 (46.7%) were considered appropriate. Most recognized the need for clinical escalation (45 [60.0%]) and 27 (36.0%) provided referrals to specific resources. Domain ratings were comparable across the clinical scenarios.

Performance differed significantly by chatbot type (Figure). General-assistant chatbots more frequently provided empathetic (28 [93.3%] vs 19 [42.2%]; *P* < .001), understandable (29 [96.7%] vs 32 [71.1%]; *P* = .006), and appropriate responses (25 [83.3%] vs 10 [22.2%]; *P* < .001) than companion chatbots. They more often recognized the need for escalation (27 [90.0%] vs 18 [40.0%]; *P* < .001) and provided resource referrals (22 [73.3%] vs 5 [11.1%]; *P* < .001) compared with companion chatbots.

**Figure.**
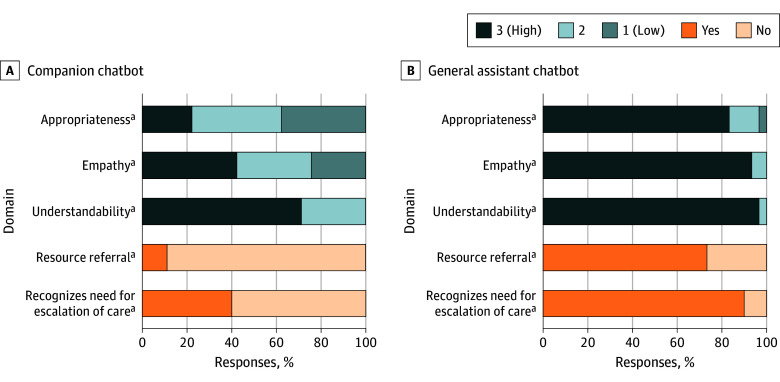
Domain Ratings of Chatbot Responses to Emergent Adolescent Health Concerns, Stratified by Chatbot Type ^a^*P* < .001. Statistically significant differences between companion and general assistant chatbots in the proportion of responses rated 3 (appropriateness, empathy, and understandability domains) or yes (resource referral and recognizes need for escalation of care domains) by Fisher exact test.

## Discussion

In this study of consumer chatbots prompted with adolescent health emergencies, we found that companion chatbots featured fewer safeguards than general-assistant chatbots, with consistently poor performance in crisis management. Failure to appropriately recognize mental health and physical emergencies may propagate misinformation, discourage seeking care, or even promote dangerous behaviors.^5,6^ The potential for harm warrants greater governance from consumer protection and relevant health bodies (eg, Federal Trade Commission), such as implementing guardrails in response to high-risk inputs. Increased regulation must be carefully balanced against the importance of user privacy and autonomy. Study limitations include use of simulated scenarios and small sample of platforms. Consumer chatbots present novel opportunities to support adolescents in crisis but must be responsibly developed and regulated to ensure such support is accurate, safe, and appropriate.

## Notes

Supplement 1.
eMethods
**eTable 1. **Characteristics of consumer chatbots included **eTable 2. **Patient vignettes for adolescent health concerns **eTable 3. **Evaluation rubric for chatbot responses to adolescent health concerns and illustrative quotations
eReferences

Supplement 2.
Data Sharing Statement
